# Cellulose Nanocrystals-Stabilized Bio-Based Waterborne Polyhydroxyurethane Nanocomposites with Enhanced Adhesive Performance

**DOI:** 10.1021/acsapm.5c03679

**Published:** 2025-12-13

**Authors:** Hsin-Chen Chen, Gilles Sèbe, Thomas Vidil, Lars. A. Berglund, Audrey Llevot, Henri Cramail, Qi Zhou

**Affiliations:** † Division of Glycoscience, Department of Chemistry, School of Engineering Sciences in Chemistry, Biotechnology and Health, KTH Royal Institute of Technology, AlbaNova University Centre, SE-106 91 Stockholm, Sweden; ‡ 359047 Université de Bordeaux, CNRS, Bordeaux INP, LCPO, UMR 5629, F-33600 Pessac, France; § Department of Fibre and Polymer Technology, 7655 KTH Royal Institute of Technology, Teknikringen 56, SE-100 44 Stockholm, Sweden

**Keywords:** waterborne polyhydroxyurethane, cellulose nanocrystals, suspension polymerization, pressure-sensitive adhesives, lap-shear strength

## Abstract

Polyurethanes are widely used in adhesive applications, but their conventional synthesis relies on hazardous isocyanates and often solvent-based formulations. In this work, waterborne polyhydroxyurethanes (PHUs) were synthesized from 1,6-hexanediol bis­(cyclic carbonate) and bio-based Priamine 1075 via catalyst-free suspension polymerization in water. Pristine cellulose nanocrystals (CNCs) acted as the sole stabilizers, eliminating the need for petroleum-derived surfactants while simultaneously serving as reinforcing nanofillers. Stable monomer-in-water emulsions were obtained with CNC loadings up to 200 mg mL^–1^ per monomer, corresponding to ∼17 wt % CNCs in the final dried nanocomposites. In the latex state, CNCs were located at the particle surfaces, ensuring colloidal stability, while in the dried PHU/CNC nanocomposites they were uniformly distributed throughout the matrix, yielding adhesives with markedly enhanced performance. The nanocomposites exhibited up to 680% and 340% increases in probe tack adhesion strength and lap-shear strength, respectively, compared with surfactant Tween 80-stabilized waterborne PHUs, reaching performance levels comparable to commercial pressure-sensitive adhesives. These findings demonstrate that combining bio-based monomers with CNC stabilization offers a robust strategy for producing sustainable, high-performance PHU adhesives consistent with green chemistry principles.

## Graphical abstract

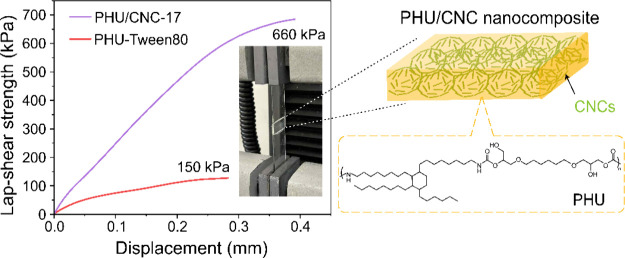

## 1 Introduction

Driven by increasingly strict regulations on volatile organic compound emissions, waterborne polyurethanes (PUs) have emerged as competitive alternatives to solvent-borne systems. Waterborne PUs also overcome the viscosity limitations associated with molten precursors in solvent-free formulations, making them particularly attractive for adhesives and coatings.^1^ In parallel, the use of isocyanate has come under regulatory control under frameworks such as REACH, further motivating the development of waterborne nonisocyanate polyurethanes (NIPUs) as safer and more sustainable coating and adhesive materials. However, direct implementation of NIPU formulations in water remains challenging due to the limited availability of water-soluble monomers.^2^ To address this, hydrophilic groups are often introduced into the polymer backbone to promote dispersibility in water. One approach has involved the synthesis of carboxylic acid-functional poly­(cyclic carbonate)­s, which become water-dispersible after neutralization with tertiary amines, followed by thermal curing with (cyclo)­aliphatic diamines to produce waterborne NIPU coatings.^3^ Another strategy has utilized amino-terminated NIPU oligomers containing tertiary amines and carboxylic acid groups, which can be dispersed in water and subsequently cross-linked with epoxy dispersions as hardeners.^4,5^ Despite enabling aqueous processing, these methods generally result in inferior water resistance, mechanical properties, and coating durability compared with solvent-based counterparts, primarily due to the incorporation of hydrophilic moieties.^4,6^

The mini-emulsion process offers an alternative route for producing waterborne NIPUs from hydrophobic monomers, reducing the need for direct modification of polymer chains. In this approach, cyclic carbonate and diamine monomers have been polymerized in mini-emulsion dispersions using surfactants such as polyethylene glycol sorbitan monooleate (Tween 80) and sodium dodecyl sulfate (SDS) to produce waterborne polyhydroxyurethanes (PHUs).^7^ Waterborne hybrid dispersions, such as poly­(hydroxyurethane)–poly­(butyl methacrylate) hybrid latexes have also been prepared via mini-emulsion method and stabilized with alkyldiphenyloxide disulfonate (Dowfax 2A1).^8^ The mini-emulsion technique broadens the range of accessible monomers and hybrid systems, while simultaneously enhancing the hydrophobicity of the resulting materials. However, residual surfactants in the latexes can migrate over time, adversely affecting optical, mechanical, and adhesive properties.^9^

Pickering emulsions are stabilized by solid particles rather than surfactants. They offer excellent stability and high stabilization efficiency while requiring only small amounts of stabilizers.^10^ Among the available particle stabilizers, cellulose nanocrystals (CNCs) derived from renewable lignocellulosic sources have proven to be effective and sustainable options for stabilizing oil-in-water emulsions and waterborne polymer latexes.^11–13^ CNCs are typically obtained through sulfuric acid hydrolysis of natural cellulose and consist of high crystalline rod-like nanoparticles with lengths of 100–200 nm and widths of 3–20 nm. Sulfate groups introduced during hydrolysis (0.6–1.1% sulfur by mass) impart negative surface charges, ensuring excellent colloidal stability in aqueous media.^14^ Their amphiphilicity and partial wettability in both oil and water phases make CNCs suitable as sustainable stabilizers for emulsions and latexes. In addition, their high surface area, low density, and high specific Young’s modulus enable CNCs to function not only as stabilizers but also as reinforcing nanofillers and functional additives in nanocomposite materials.^15^

CNCs have also been reported as rheological modifiers in CNCs-mineral oil Pickering emulsions.^16^ CNCs-stabilized emulsions show greater stiffness, viscosity, and shear stability compared with surfactant-stabilized emulsions, attributed to their high specific area, stiffness, and abundance of hydroxy groups that enable extensive hydrogen bonding. CNCs-based high internal phase Pickering emulsion (HIPPE) with 80% internal phase was successfully prepared at 0.5 wt % CNC concentration, demonstrating shear-thinning behavior, high solid viscoelasticity, and good storage stability.^17^ These properties make CNC-stabilized HIPPE suitable for 3D printing, producing promising nontoxic materials for biomedical tissue engineering. CNCs have been used as stabilizers to produce CNC-waterborne polymer nanocomposites where CNC and the polymer matrix are combined during the emulsification process in water. This approach facilitates uniform filler dispersion, which is often difficult to achieve by conventional methods. CNC-PLA nanocomposites prepared using the Pickering emulsion approach showed CNC network formation within the PLA matrix, resulting in enhanced storage modulus, tensile modulus, flexural strength and modulus.^18^

The aim of this study was to use pristine CNCs as the sole stabilizers for waterborne PHUs in order to improve sustainability as well as mechanical and adhesive performances (Figure 1). Stable latexes were obtained by catalyst-free polyaddition of 1,6-hexanediol bis­(cyclic carbonate) (HCC) and bio-based Priamine 1075 in water, where CNCs provided droplet stabilization without surfactants. In this suspension polymerization, CNCs stabilized the monomer-in-water droplets and, after polymerization, were located on the surfaces of the PHU latex particles. Upon drying, they became uniformly distributed within the PHU matrix, enabling CNC loadings up to 17 wt % in the final nanocomposites. The hydroxy groups of PHUs offered additional opportunities for hydrogen bonding with CNCs, enhancing interfacial interactions and adhesion. Achieving such high CNC contents in polymer nanocomposites while maintaining their reinforcing effects is typically challenging. Here, we systematically investigated how CNC concentration affects monomer droplet size, PHU latex morphology, and the thermal and adhesive properties of the dried nanocomposites. The resulting PHU/CNC nanocomposites clearly demonstrated the dual role of CNCs, serving not only as stabilizers in latex but also as reinforcing nanofillers and adhesion promoters in the dried materials, and the supervisor adhesive functionality, extending their potential application scope.

**Figure 1:**
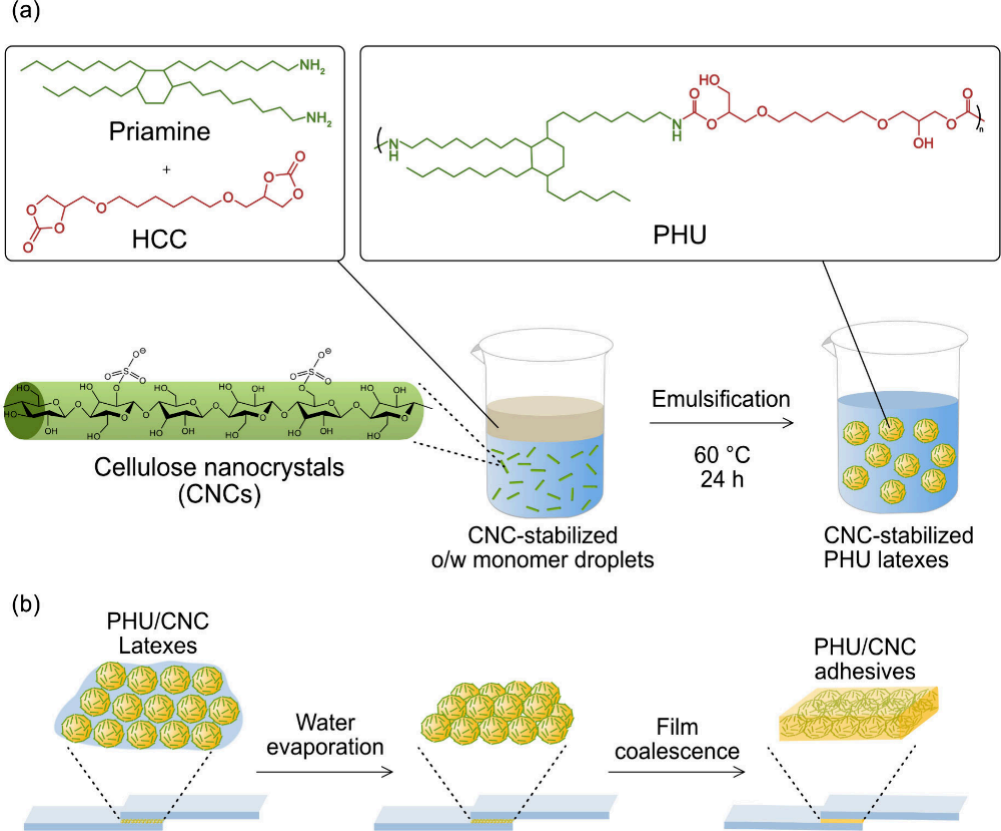
Schematic illustration of waterborne PHU synthesis and application. (a) Chemical structures and reaction scheme of monomers (Priamine and HCC), CNC-stabilized oil-in-water (o/w) emulsification process leading to PHU latexes, and (b) demonstration of PHU/CNC nanocomposite adhesive application.

## 2 Experimental Section

### 2.1 Materials

1,6-Hexanediol diglycidyl ether was purchased from Biosynth (Staad, Switzerland). 1,6-hexandiol bis­(cyclic carbonate) was synthesized by CO_2_ carbonation of 1,6-hexanediol diglycidyl ether according to our previously reported procedure.^19^ Priamine 1075 was supplied by Croda (New Jersey, USA) and used as received. Sodium chloride (NaCl), tetrabutylammonium iodide (TBAI), and Tween80 were purchased from Merck KGaA (Darmstadt, Germany) and used without further purification. CNCs derived from wood cellulose were obtained from the University of Maine (Orono, ME, USA) in the form of a 12 wt % water suspension. Prior to use, the CNC suspension was diluted to 1 wt % and dialyzed against deionized water.

### 2.2 Preparation of Monomer-in-Water Droplets and PHU Latexes

To prepare the organic phase, HCC and Priamine were mixed at room temperature, with the total monomer concentration fixed at 10 wt % relative to the total emulsion. The aqueous phase was prepared separately by dispersing CNCs in deionized water using tip-sonication (Branson SFX550 Sonifier). Sodium chloride (75 mM) was added to partially screen the CNC surface charges. The organic phase was then introduced into the CNCs dispersion and emulsified at 15000 rpm for 15 s using an IKA T 25 digital Ultra-Turrax, generating monomer-in-water droplets. The emulsion droplets were polymerized at 60 °C for 24 h under continuous mechanical stirring at 200–300 rpm, followed by cooling the resulting latexes to room temperature. The obtained PHU latexes were stored at ambient conditions until further analysis.

### 2.3 Adhesion Testing

Adhesion properties of the CNCs-stabilized PHU latexes were first evaluated by probe-tack tests. Prior to testing, latexes were cast on Teflon Petri dishes, dried at room temperature, and collected. Tack tests were performed on a Discovery HR 20 rheometer (TA Instruments) using an 8 mm stainless steel parallel plate geometry with a Peltier plate temperature control. The dried latexes were mounted on the bottom plate, and the top probe was brought into contact under a load of 4.9 N. The probe was then retracted from the adhesive surface at a constant rate of 0.1 mm s^–1^, corresponding to a strain rate of 1 s^–1^. For each sample, three replicate measurements were conducted.

Single-lap shear tests were also performed to further assess adhesion strength. The prepared 10 wt % PHU latexes were concentrated to 30 wt % by centrifugations. Two glass slides (76 × 26 × 1 mm^3^) were used as adherents, and 80 μL of the concentrated latex (corresponding to ∼20 mg dried sample) was applied to one end of the bottom slide. A second slide was placed on top, leaving an overlapping adhesion area of 26 × 10 mm^2^. To ensure consistent joint formation, an additional glass slide was placed underneath the top slide, and five assemblies were pressed between two aluminum plates under a 2 kg load. The joints were dried at 60 °C for 24 h and then separated from the supports. Lap shear tests were performed on an Instron universal testing machine equipped with a 500 N load cell at an extension rate of 1 mm min^–1^. Lap shear strength was calculated as the maximum shear force divided by the bonded area.

### 2.4 Characterizations

Droplet and latex morphologies were analyzed by confocal laser scanning microscopy (Zeiss LSM 800, Carl Zeiss AG, Oberkochen, Germany) equipped with an Airyscan detection unit, and the acquired images were processed using ZEN Blue 2.1 software. Droplet and latex size distributions were determined using a MasterSizer 3000 granulometer (Malvern), with samples dispersed in deionized water in the manual dispersion chamber. The chemical structures of the PHUs were investigated by ^1^H NMR spectroscopy (Bruker Advance 400 MHz) using CDCl_3_ as the solvent. Molecular weights were determined by size exclusion chromatography (SEC) in chloroform containing triethylamine at 30 °C (flow rate: 0.8 mL min^–1^) using Agilent PLgel columns (5 μm Guard, 7.5 × 50 mm; Mixed-C, 7.5 × 300 mm). Glass transition temperature (*T*_g_) was measured by differential scanning calorimetry (DSC, Mettler Toledo) at a heating rate of 10 °C min^–1^ under a nitrogen flow of 50 mL min^–1^. Two heating cycles from −80 to 150 °C, with a cooling cycle in between, were performed, and *T*_g_ values were taken from the second heating cycle. The Attenuated Total Reflectance Fourier Transform Infrared (ATR-FTIR) spectra of CNCs, neat PHU, and PHU/CNC nanocomposites were recorded using a Spectrum 2000 spectrometer (PerkinElmer) over the range of 4000–600 cm^–1^ with a resolution of 4 cm^–1^. Field-emission scanning electron microscopy (FESEM) was conducted using a Hitachi S-4800 instrument. Neat PHU and PHU/CNC nanocomposite samples were dried on plasma-treated silica wafers and sputter-coated with a thin Pt/Pd layer prior to imaging.

## 3 Results and Discussion

### 3.1 Optimization of CNCs-Stabilized Monomer Droplets

Stable monomer droplets are prerequisites for successful suspension polymerization process. Moreover, monomer droplet sizes determine their final latex sizes.^20^ Therefore, the investigation and optimization of CNC stabilization on monomer droplets was considered a primary objective. To facilitate the formation of stable CNCs-stabilized monomer-in-water emulsions, NaCl was introduced to partially screen the negative charges on CNC surfaces. This ensured a sufficient charge density to facilitate droplet stabilization while preventing excessive electrostatic repulsion between CNCs that would otherwise destabilize the system. NaCl concentrations between 0 and 75 mM were examined. Emulsions did not form effectively at concentrations below 15 mM, whereas phase separation with unstabilized monomers was observed for NaCl concentrations between 30 and 60 mM (Figure S1, Supporting Information). Full stabilization was achieved at 75 mM NaCl, which was therefore adopted in all subsequent formulations.

The mass of CNCs per monomer volume (*m*_*p*_) was also optimized. A minimum of 50 mg CNCs per mL of monomers (HCC/Priamine mixture) was required to stabilize the droplets. Accordingly, *m*_*p*_ was varied between 50 to 300 mg mL^–1^ at a fixed HCC/Priamine molar ratio of 1:1 and a monomer-to-water ratio of 10:90 by weight. Following droplet generation using an Ultra-Turrax disperser, the obtained emulsions were kept at 22 °C for 3 h. Before size analysis, the stabilized droplets were redispersed using a vortex mixer. Confocal laser scanning microscopy images of the HCC/Priamine emulsion droplets are shown in Figure 2, and their size distributions and the inverse average D­[3,2] Sauter’s diameters determined by MasterSizer 3000 granulometer are presented in Figure 3. Unimodal droplet size distributions were observed for all CNC loadings. Increasing the CNCs concentration led to a systematic decrease in droplet size, as evidenced by both the reduced average diameters and the transition from coarse to fine droplet morphologies observed in confocal laser scanning microscopy images (Figure 2). At CNCs concentrations above 250 mg mL^–1^, some droplets displayed irregular shapes, which can be attributed to the increased viscosity of the water phase at high CNC contents. Under these conditions, the dispersed droplets were unable to fully relax into spherical morphologies.

**Figure 2:**
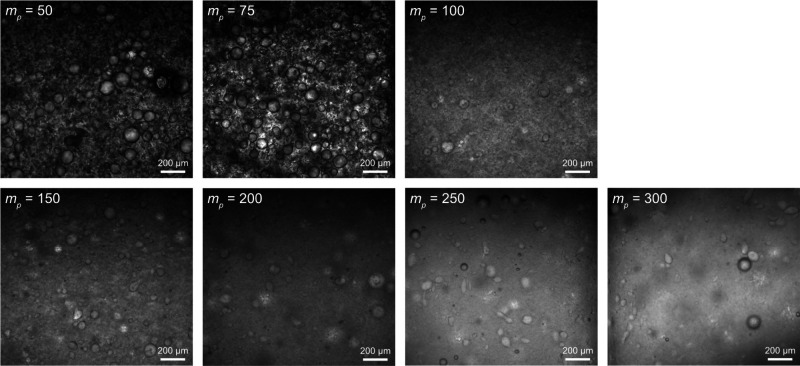
Confocal laser scanning microscopy images of HCC/Priamine emulsion droplets stabilized with CNCs at *m*_*p*_ values of 50, 75, 100, 150, 200, 250, and 300 mg mL^–1^ (scale bar: 200 μm).

**Figure 3:**
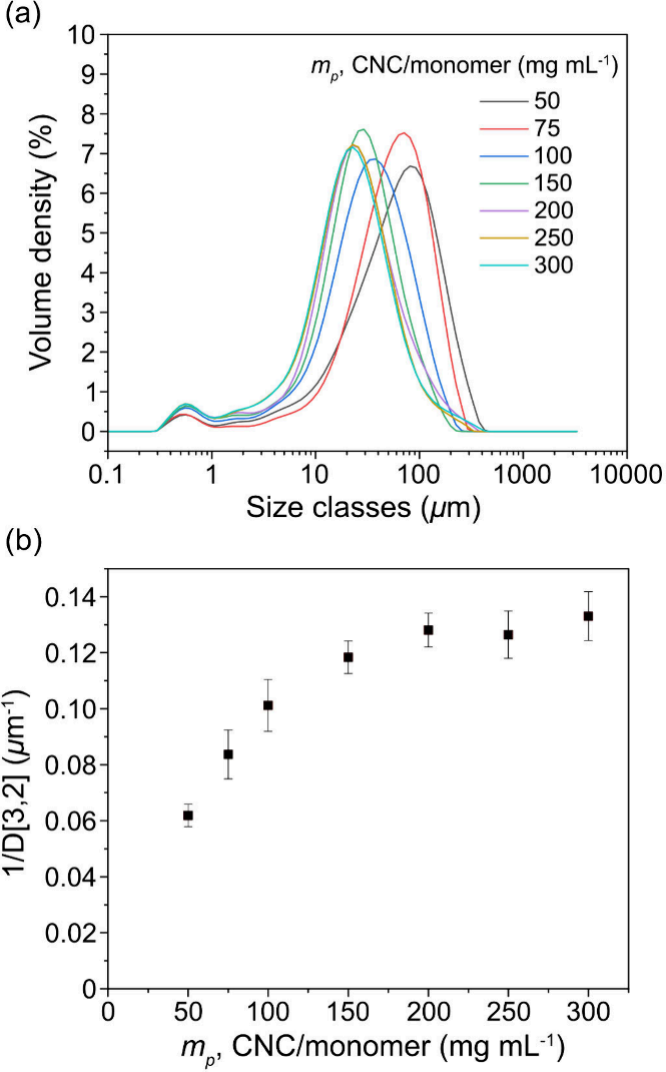
(a) Droplet size distributions of HCC/Priamine emulsions stabilized with CNCs at different concentrations and (b) reciprocal Sauter mean diameter (1/D­[3,2]) as a function of CNC mass per monomer volume (*m*_*p*_, 50–300 mg mL^–1^).

The reciprocal Sauter mean diameter (1/D­[3,2]) was plotted as a function of CNCs concentration (Figure 3b). A linear relationship was observed between 0 and 100 mg mL^–1^ of CNCs per monomer mixture, indicating that at lower loadings the CNC nanoparticles were insufficient to stabilize the droplets immediately after agitation (shearing). Consequently, coalescence occurred until a stable droplet size was reached, reducing the overall interfacial area. At concentrations above 150 mg mL^–1^, the droplet size became independent of CNC content, suggesting that the available nanoparticles were sufficient to stabilize the droplets directly upon formation. Such a plateau regime is consistent with the trends reported in Pickering emulsion systems.^21,22^ For the HCC/Priamine system investigated here, the plateau droplet size was reached at a CNCs concentration of 200 mg mL^–1^, giving a D­[3,2] diameter of 7.1 ± 1.1 μm.

### 3.2 CNCs-Stabilized PHU Latex Formation

Our earlier work demonstrated that CNCs can stabilize HCC/siloxane amine droplets at the oil–water interface and enable efficient polyaddition between cyclic carbonates and amines via suspension polymerization in water.^19^ In the present study, the same approach was applied under catalyst-free conditions, with polymerizations carried out at 60 °C for 24 h under constant mechanical stirring (200 rpm), using CNCs as the sole stabilizers.

For initial trials, a CNC *m*_*p*_ value of 100 mg mL^–1^ was selected to confirm successful latex formation. This CNC concentration corresponds to the transition between the linear and plateau regions of the droplet size as a function of CNC concentration as shown in Figure 3, ensuring sufficient stabilization during polymerization while maintaining uniform spherical morphologies at moderate viscosity. In addition, the effect of the HCC/Priamine ratio on monomer conversion was investigated. Given that partial hydrolysis of cyclic carbonates can occur in aqueous environment, optimizing the monomer ratio is essential to maximize amine conversion.^19^ Therefore, HCC/Priamine ratios of 1.0, 1.2, and 1.3 were examined while fixing the CNC *m*_*p*_ at 100 mg mL^–1^. To determine the monomer conversion, the resulting PHUs were characterized by ^1^H NMR spectroscopy in CDCl_3_ (Figure 4). The molecular weight of the PHUs were further assessed by SEC (Figure S2a, Supporting Information) and the results are summarized in Table 1.

**Table 1:** Molecular Weight and Amine Conversion of PHUs with Varied HCC/Priamine Ratios and Varied CNC *m*_*p*_ (mg mL^–1^)­^a^

HCC/Priamine ratio	CNC *m*_*p*_ (mg mL^–1^)	$\overset{®}{M_{n}\;}$	$\overset{®}{M_{w}\;}$	Đ	Amine conversion (%)
1.0	100	4200	9100	2.2	81.9
1.2	100	4500	9500	2.1	91.1
1.3	100	4400	9400	2.2	89.7
1.2	150	4300	9100	2.1	87.0
1.2	200	3900	8400	2.2	84.4
1.2	^a^	4700	11900	2.6	90.1

a $\overset{®}{M_{n}\;}$ and $\overset{®}{M_{w}\;}$ determined by using polystyrene standards on SEC and amine conversion calculated from ^1^H NMR spectra.

b Stabilized by Tween 80.

**Figure 4:**
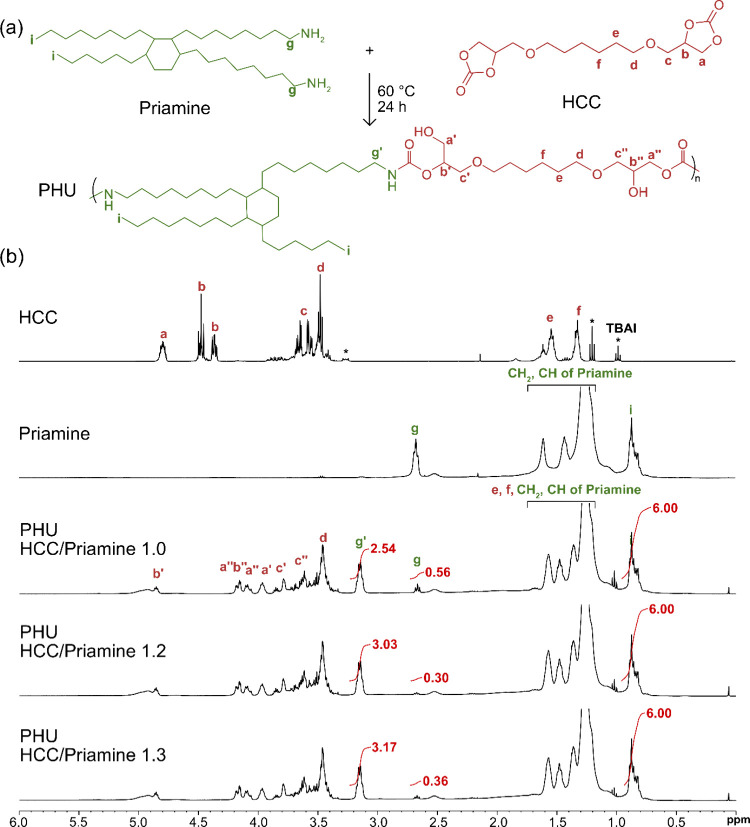
(a) Reaction between HCC and Priamine for PHU synthesis. (b) ^1^H NMR spectra of HCC, Priamine, and dried PHU latexes prepared at HCC/Priamine ratios of 1.0, 1.2, and 1.3 with a fixed CNC *m*_*p*_ of 100 mg mL^–1^ in CDCl_3_.

A complete conversion of cyclic carbonates to PHUs was confirmed by the disappearance of the characteristic cyclic carbonate peaks **a** and **b** across all HCC/Priamine ratios (Figure 4). Success of aminolysis and PHUs formation were further evidenced by the appearance of new resonances corresponding to carbamate protons: **b′** at δ = 4.86 ppm, **a″** and **b″** at δ = 4.18–4.16 ppm, **a″** at δ = 4.10 ppm, **a′** at δ = 3.97 ppm, **c′** at δ = 3.84 ppm, **c″** at δ = 3.62 ppm, and **g′** at δ = 3.15 ppm. Residual amines were detected in all formulations by the presence of peak **g** at δ = 2.67 ppm, corresponding to the proton in the α-position of the amine group. Notably, this peak showed the lowest intensity at HCC/Priamine 1.2. Amine conversion was quantified from the integration ratio of peaks **g** and **g′**. Increasing the HCC/Priamine ratio from 1 to 1.2 improved amine conversion by nearly 10% (from 81.9 to 91.1%). Further increasing the ratio to 1.3 did not achieve additional improvement. Consistent with these results, the highest number-average molecular weight $(\overset{®}{M_{n}\;}$ = 4500 g mol^–1^), weight-average molecular weight $(\overset{®}{M_{w}\;}$ = 9500 g mol^–1^), and dispersity (Đ = 2.1) were obtained at a HCC/Priamine ratio of 1.2, indicating this ratio as optimal for the organic phase of the CNC-stabilized PHU latexes (Table 1).

To evaluate the effect of CNC loading, the CNC *m*_*p*_ was varied from 100 to 200 mg mL^–1^ while maintaining the optimized HCC/Priamine ratio of 1.2. Increasing CNC concentration reduced droplet sizes from D­[3,2] diameter of 10.2 ± 0.8 to 7.9 ± 0.6 μm (Figure 5a), consistent with the trend observed at a HCC/Priamine ratio of 1.0 (Figure 3a). The successful formation of PHU latexes at all CNC concentrations was further confirmed by the fluorescence observed in confocal laser scanning microscopy images under 405 nm excitation (Figure 6). The fluorescence originates from aggregated carbamate groups, which induce clusterization-triggered emission (CTE).^19^ Latexes prepared with CNC *m*_*p*_ of 150 and 200 mg mL^–1^ showed broader size distributions with shoulder peaks at larger diameters compared to the narrower, unimodal distribution observed at 100 mg mL^–1^ (Figure 5b). This effect can be attributed to the significant higher viscosity of the aqueous phase at elevated CNC content. Under these conditions, maintaining droplet stability during suspension polymerization becomes more difficult, leading to occasional coalescence and larger particle formation in the latex. Indeed, stable latex formation at CNC *m*_*p*_ of 200 mg mL^–1^ could be reached by an increased stirring speed of 300 rpm compared to 200 rpm for lower concentrations.

**Figure 5:**
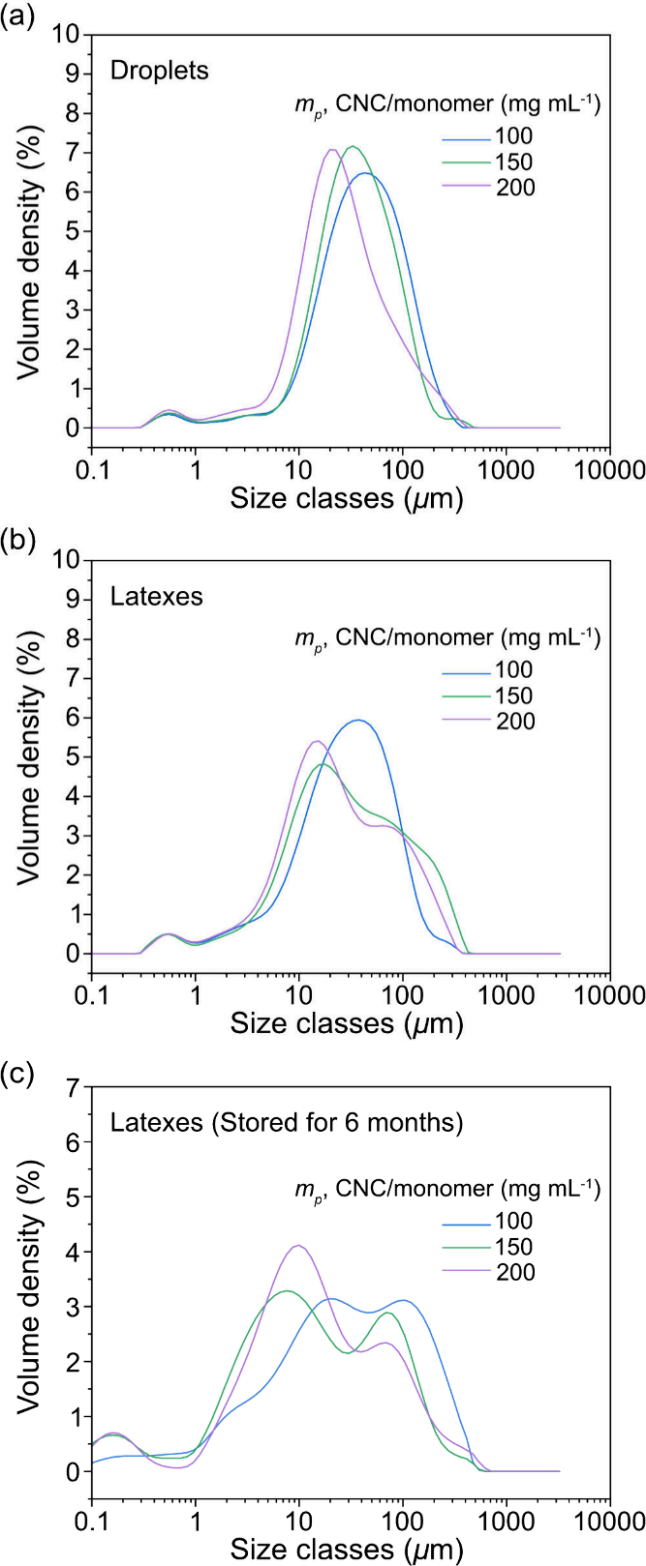
Size distributions of CNCs-stabilized (a) HCC/Priamine droplets and (b) PHU latexes prepared with CNC *m*_*p*_ of 100, 150, and 200 mg mL^–1^ at a fixed HCC/Priamine ratio of 1.2, and (c) the latexes stored for 6 months after preparation.

**Figure 6:**
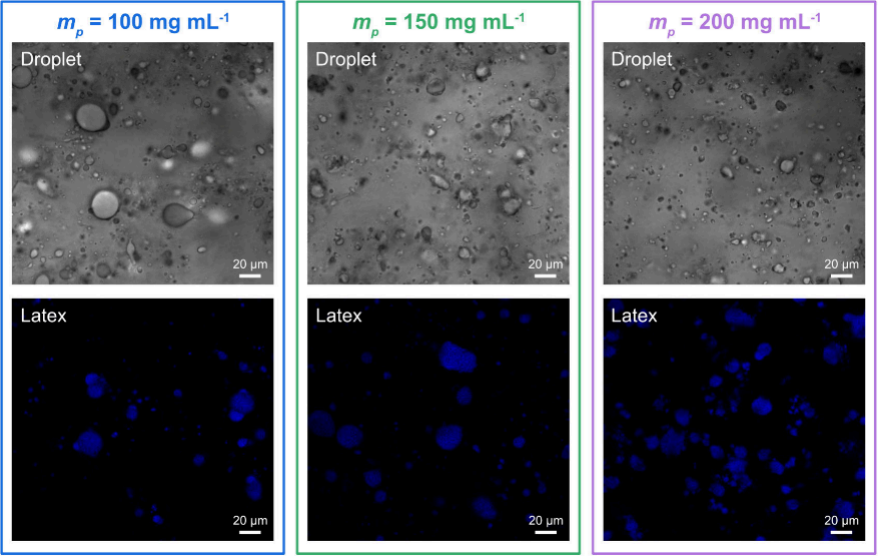
Confocal laser scanning microscopy images of HCC/Priamine droplets and the corresponding PHU latexes prepared with CNC *m*_*p*_ of 100, 150, and 200 mg mL^–1^ at a fixed HCC/Priamine ratio of 1.2 (scale bar: 20 μm).

A slight decrease in conversion from 91% to 84% was also observed when increasing CNC *m*_*p*_ from 100 to 200 mg mL^–1^ (Table 1; Figure S2b and Figure S3, Supporting Information). Similar viscosity-driven effects have been reported in Pickering emulsions stabilized by CNCs, as well as in suspension polymerization systems, where higher stabilizer concentrations often lead to irregular droplet morphologies, broader particle size distributions, and reduced conversion efficiencies.^23,24^ These observations indicate that CNC concentration must be carefully optimized to achieve effective PHU latex synthesis by balancing stabilization efficiency with water phase viscosity.

The long-term colloidal stability of CNC-stabilized PHU latexes was evaluated after six months of storage. As shown in Figure S4, Supporting Information, latexes prepared with CNC *m*_*p*_ of 100, 150, and 200 mg mL^–1^ maintained their macroscopic appearance over time, exhibiting no visible aggregation, phase separation, or sedimentation compared to the freshly prepared samples. All dispersions remained free-flowing, indicating that CNC-based interfacial structures provided sufficient steric and electrostatic stabilization to resist destabilization during prolonged storage. The potential changes in particle size were further analyzed (Figure 5c), the latexes stabilized with higher CNC concentrations (150 and 200 mg mL^–1^) retained their original size profiles, showing minimal changes in distribution shape or peak position. This stability reflects the stronger resistance to coalescence imparted by the higher particle coverage and increased continuous-phase viscosity at elevated CNC contents. In contrast, the latex stabilized at 100 mg mL^–1^ exhibited a broader, partially bimodal distribution with a noticeable shift toward larger particle sizes, indicating gradual coalescence during storage. These results suggest that while 100 mg mL^–1^ of CNC is sufficient for initial latex formation, long-term stability benefits significantly from higher CNC loadings that provide more robust interfacial protection.

### 3.3 From Latexes to PHU/CNC Nanocomposites

To further investigate the reinforcing role of CNCs in the PHU/CNC nanocomposites, the CNC-stabilized PHU latexes were dried at room temperature to produce solid coatings. CNCs contents of 9, 13, and 17 wt % were achieved from latexes with CNCs *m*_*p*_ of 100, 150, and 200 mg mL^–1^, respectively, and are denoted as PHU/CNC-9, PHU/CNC-13, and PHU/CNC-17. The thermal properties of the nanocomposites were evaluated by DSC (Figure S5, Supporting Information). All PHU/CNC nanocomposites exhibited glass transition temperatures (*T*_*g*_) between −26 °C and −20 °C. A systematic increase in *T*_*g*_ was observed with increasing CNCs content, with a shift of ∼6 °C as the CNCs content increased from 9 to 17 wt %. The trend is consistent with previous reports of CNCs-reinforced polymeric nanocomposites, where higher *T*_*g*_ values were attributed to good compatibility and hydrogen-bonding interactions between CNCs (or poly­(3-hydroxybutyrate-co-3-hydroxyvalerate)-grafted CNCs) and the host polymer matrices.^25,26^ These results suggest that CNCs restrict PHU chain mobility through a combination of physical reinforcement and possible hydrogen bonding with pendant hydroxy and urethane groups in the matrix.^26,27^

Direct evidence for hydrogen bonding between CNCs and the PHU matrix was obtained from ATR-FTIR analysis (Figure S6, Supporting Information). The urethane carbonyl region showed clear, systematic changes when comparing PHU/CNC nanocomposites with the surfactant-stabilized PHU-Tween80 reference. In PHU-Tween80, two distinct carbonyl bands were observed: a hydrogen-bonded C=O peak at 1698 cm^–1^ and a nonbonded urethane C=O peak at 1723 cm^–1^. Upon incorporation of CNCs, the hydrogen-bonded carbonyl band became broader and shifted slightly toward lower wavenumbers, reflecting strengthened or additional hydrogen-bonding interactions. Concurrently, the intensity of the nonbonded C=O band at 1723 cm^–1^ decreased progressively with increasing CNC content, indicating that a greater fraction of urethane groups became hydrogen-bonded in the presence of CNCs. These spectral changes, together with the broadened O–H/N–H stretching region (3600–3000 cm^–1^), confirm that CNCs actively participate in hydrogen bonding with the PHU matrix.^28–30^ Such interactions are consistent with the observed increases in *T*_*g*_ and the enhanced mechanical performance of PHU/CNC nanocomposites.

The morphological characteristics of the PHU composites provide further insight into the distinct roles of CNC and Tween 80 during suspension polymerization. In the Tween 80-stabilized PHU (Figure 7a), numerous spherical features are visible on the film surface. These structures are attributed to micellar or micelle-derived aggregates of Tween 80 rather than polymer domains. Tween 80 is known to form micelles with a hydrodynamic diameter of ∼10 nm in water,^31^ but significantly larger micellar-type aggregates (60–70 nm) have been reported in nonpolar organic media at concentrations above 7 mM.^32^ The sizes and morphology observed after drying Tween80-stabilized PHU are consistent with such surfactant-rich aggregates. In contrast, the PHU/CNC-17 nanocomposite (Figure 7b) exhibited a continuous, fibrillar surface densely covered with rod-like CNCs. The CNCs featured lengths in the range of ∼150–300 nm, consistent with the dimensions reported previously. The CNCs had an average width of 5.9 ± 1.5 nm and an average length of 201.9 ± 70.7 nm, as determined by atomic force microscopy (AFM) in our previous work.^19^ These observations confirm that CNCs remain dispersed as individual nanorods within the polymer matrix even at the high loading of 17 wt %. No clustered CNC domains or particulate aggregates were detected, indicating that CNC-stabilized suspension polymerization effectively prevents nanoparticle flocculation during both droplet stabilization and PHU formation. Notably, lower magnification images showed surface buckling and shrinkage features for the CNCs-stabilized PHU (Figure S7, Supporting Information). However, these were artifacts of PHU soft matrix rather than CNC aggregates. The absence of such ambiguity in the present high-magnification image (Figure 7b) clarifies the nanoscale distribution of CNCs. These results confirm that CNCs are well integrated into the PHU network at high loading levels, supporting their role as both stabilizers during polymerization and reinforcing nanofillers in the final composite.

**Figure 7:**
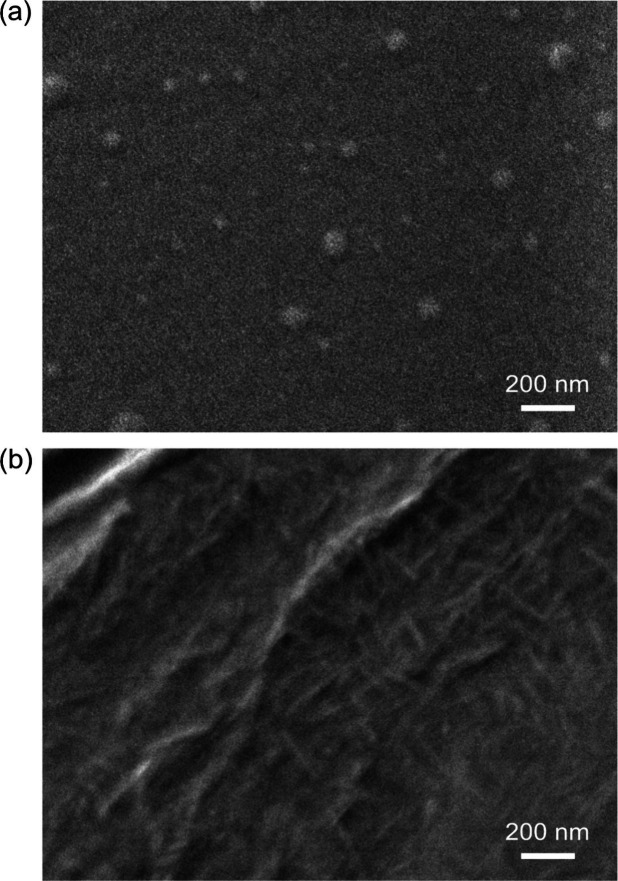
FESEM surface images of (a) Tween80-stabilized PHU and (b) PHU/CNC-17 nanocomposite, showing uniform CNCs distribution without visible aggregation at high loading.

### 3.4 Probe Tack Performance

The adhesion behavior of PHU/CNC nanocomposites was investigated by probe tack tests to evaluate their potential as adhesive materials and to assess the effect of CNC content on performances (Figure 8). As a control, PHU latex stabilized with the nonionic surfactant Tween80 was prepared for comparison and coded as PHU-Tween80. All samples showed cohesive failure and filament formation upon detachment, indicating strong internal cohesion. Compared to PHU-Tween80, CNCs-reinforced systems exhibited substantial and synergistic improvements across all tack parameters, including peak strength (σ_*max*_), work of adhesion (*W*_*adh*_), and maximum extension (ε_*Max*_), as summarized in Table S1, Supporting Information. At 9 wt % CNCs, σ_*max*_ increased by 335%, while *W*_*adh*_ improved by more than 300% compared to the surfactant-stabilized system. These enhancements are attributed to the reinforcing effect of CNCs, which increased the elasticity of the adhesive network. Higher elasticity has been correlated with increased σ_*max*_ values, as σ_*max*_ is associated with cavity growth and local elastic deformation.^33^ At higher CNC contents, the reinforcing effect was even more pronounced. With 17 wt % CNCs, σ_*max*_ increased by 680% compared to PHU-Tween80. Notably, this high CNC loading did not compromise extensibility or filament formation. PHU/CNC-17 achieved highest *W*_*adh*_ of 46.4 J/m^2^, indicating the combined benefits of reinforcement and extensibility. This *W*_*adh*_ value is comparable to the previously studied conventional isocyanate-based Waterborne PUs.^34,35^

**Figure 8:**
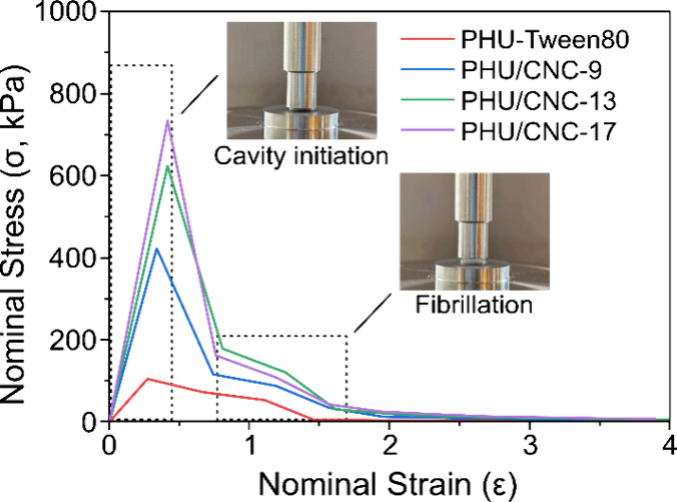
Stress–strain curves from probe-tack tests of CNC-stabilized PHU nanocomposites at different CNC loadings compared with surfactant-stabilized PHU (PHU-Tween80). Inset photographs show representative probe-tack images illustrating cavity initiation and fibrillation.

The enhancement of adhesion properties by CNC addition has been reported on CNC-reinforced polymeric pressure sensitive adhesives (PSAs).^36–38^ The improvement was attributed to hydrogen bonding between CNCs and the polymer matrix, along with the increased hydrophilicity from CNCs surface hydroxy groups.^39,40^ In the present system, reinforcement was further supported by hydrogen bonding between CNCs and the pendant hydroxy and urethane groups in PHU, in addition to interactions between CNCs. Unlike previous studies,^39,41^ no premature debonding or insufficient fibrillation was observed, even at 17 wt % CNCs. This suggests that CNCs remained uniformly distributed within the PHU matrix, as confirmed by SEM analysis (Figure 7).

### 3.5 Lap-Shear Adhesion Strength

The adhesion performance of PHU/CNC nanocomposites was further evaluated by single-lap shear tests (Figure 9a). Adhesive joints were prepared by applying concentrated (30 wt %) latexes onto cleaned glass substrates, followed by pressing and drying at 60 °C for 24 h. All specimens exhibited cohesive failure modes (Figure 9b), indicating strong internal cohesion as revealed by probe tack tests. Compared to PHU-Tween80 (150 kPa), incorporation of CNCs significantly increased lap-shear strength to 367, 579, and 659 kPa for PHU/CNC-9, PHU/CNC-13, and PHU/CNC-17, respectively. At 17 wt % CNCs, the adhesive joint showed up to a 340% increase in lap-shear strength and a 46% increase in displacement, demonstrating that reinforcement did not compromise ductility. Notably, the lap-shear strengths of PHU/CNC-13 and PHU/CNC-17 exceeded that of previously reported gels and hydrogels,^42–45^ elastomer and supramolecular adhesives,^46,47^ and crossed-link PSAs,^48–50^ and were comparable to commercial PSA clothing and medical tapes^51^ (Figure 9c).

**Figure 9:**
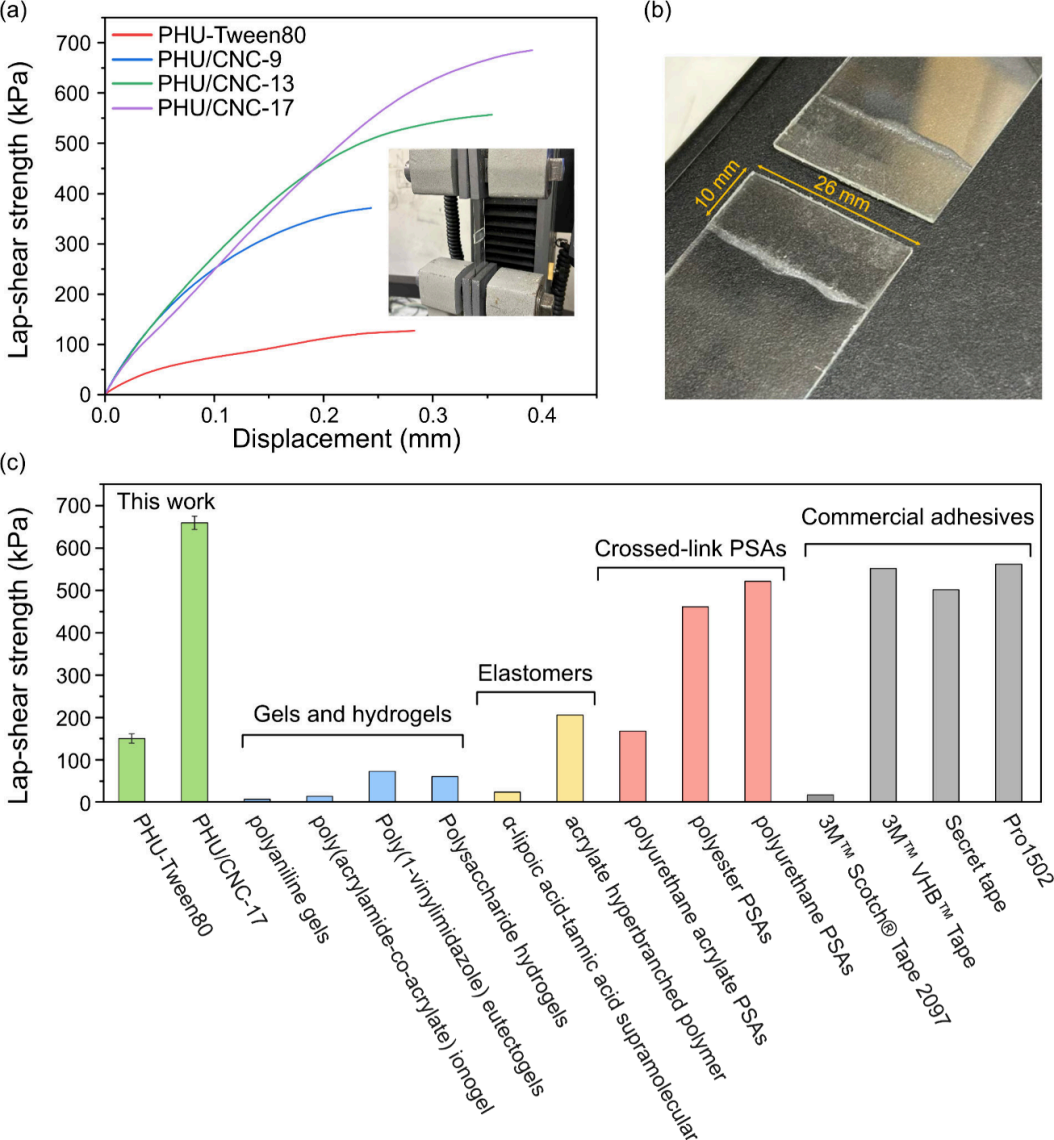
(a) Single-lap shear stress-displacement curves of Tween80-stabilized PHU and CNC-stabilized PHU nanocomposites. Photograph of experimental setup for lap-shear testing using an Instron universal testing machine. (b) Photograph of typical fracture surface of tested adhesive joint after lap-shear test. (c) Comparison of lap-shear strengths of PHU/CNC-17 composite with reported pressure sensitive adhesive systems.

Unlike most adhesives, where improvements in tack often come at the expense of shear strength,^52^ CNC simultaneously enhanced both properties. A similar trend has been reported for CNC-stabilized acrylic latex nanocomposites.^39,40^ Since the resistance of polymer chains to creep under a constant force determines the shear strength of PSAs, CNCs in PHU matrices served as physical anchoring points, enhancing chain resistance to movement and thereby increasing shear strength.^53,54^ In addition, higher CNC loadings promoted hydrogen bonding both within CNCs and between CNCs and the PHU matrix, which enhanced cohesive strength and suppressed crack propagation, consistent with earlier reports.^39,40,55^ The uniformly distributed CNCs within PHUs further contributed to the remarkable lap-shear strength improvement by enabling effective load transfer from the PHU matrix to the CNCs.^56^

Despite the strong adhesion performance achieved in this work, it is important to acknowledge the inherent limitations of the current waterborne PHU/CNC latex system. The PHU matrix used here is non-cross-linked, and therefore its absolute lap-shear strength remains lower than that of commercial nonwaterborne PU adhesives, which typically rely on extensive chemical cross-linking to achieve high structural integrity. For example, commercial two-component PU systems such as TEROSON PU 6700 and ARALDITE 2028-1 exhibit lap-shear strengths in the range of 8–10 MPa on aluminum substrates. Cross-linked PHU derived from α-alkylidene cyclic carbonates and polyamines reached lap-shear strengths of ∼11 MPa.^57^

## 4 Conclusion

Waterborne PHUs stabilized exclusively by CNCs were successfully synthesized from 1,6-hexanediol bis­(cyclic carbonate) and bio-based Priamine 1075, yielding latexes that could be dried into solid nanocomposites with adhesive functionality. Increasing CNC concentrations reduced monomer droplet size but also raised the viscosity of the aqueous phase and required higher stirring speeds to maintain stable suspensions. CNC loadings of 9, 13, and 17 wt % in PHU/CNC nanocomposites were achieved without signs of aggregation by increasing CNCs concentration when preparing CNC-stabilized PHU latexes. Owing to the reinforcing effects of CNCs and hydrogen bonding among CNCs and pendant hydroxy and urethane groups of PHU, the nanocomposites exhibited increased glass transition temperatures (*T*_g_) and substantial improvements in both tack and lap-shear adhesion compared with surfactant-stabilized PHU. In summary, CNC-stabilized suspension polymerization offers a versatile strategy for integrating high CNC contents into waterborne PHUs, enabling tunable and synergistically enhanced adhesion properties suitable for sustainable adhesive applications. This approach exemplifies how combining bio-based monomers with renewable nanofillers and surfactant-free processing can advance the development of sustainable polyurethane adhesives aligned with the principles of green chemistry.

## Supplementary Material



## Notes

NaCl concentration optimization and CNCs-stabilized monomer droplets; SEC of dried PHU latex with varied HCC/Priamine ratio and CNC concentrations; ^1^H NMR spectra of the dried PHU latex with varied CNC concentrations; latex stability after 6 months of storage; DSC thermograms; ATR-FTIR analysis; FE-SEM; summary of probe tack properties (PDF)
