# Supplementary material for: Cellulose Nanocrystals-Stabilized Bio-Based Waterborne Polyhydroxyurethane Nanocomposites with Enhanced Adhesive Performance
Source: ACS Appl Polym Mater. 2025 Dec 13;7(24):16879–89. doi: 10.1021/acsapm.5c03679 (PMC12750532; doi:10.1021/acsapm.5c03679)
Supplement: Supplementary file 1 [file ap5c03679_si_001.pdf]

**Supporting information**

# Cellulose Nanocrystals-Stabilized Bio-Based Waterborne Polyhydroxyurethane nanocomposites with Enhanced Adhesive Performance

Hsin-Chen Chen,[a,b] Gilles Sèbe,[b] Thomas Vidil,[b] Lars. A. Berglund,[c] Audrey Llevot,[b,*] Henri Cramail [b,*], and Qi Zhou,[a,*]

[a] Division of Glycoscience, Department of Chemistry, School of Engineering Sciences in Chemistry, Biotechnology and Health, KTH Royal Institute of Technology, AlbaNova University Centre, SE-106 91 Stockholm, Sweden

[b] Univ. Bordeaux, CNRS, Bordeaux INP, LCPO, UMR 5629, F-33600 Pessac, France

[C] Department of Fibre and Polymer Technology, KTH Royal Institute of Technology, Teknikringen 56, SE-100 44 Stockholm, Sweden.

E-mail: qi@kth.se, henri.cramail@enscbp.fr, audrey.llevot@enscbp.fr

Supporting Information contains 7 pages including 7 Figures and 1 Table.

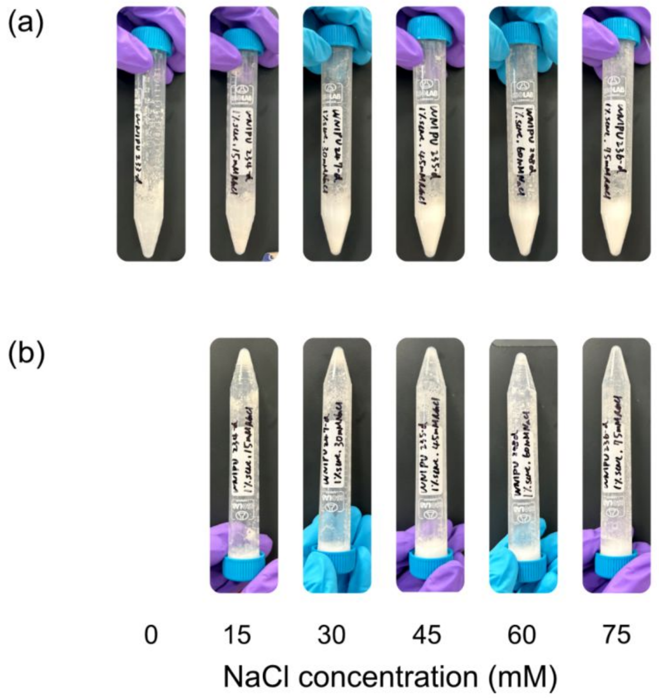


**Figure S1.** (a) HCC/Priamine monomer droplets with varied NaCl concentration of 0–75 mM and (b) the residual un-stabilized monomers observation when flipping over the falcon tube.

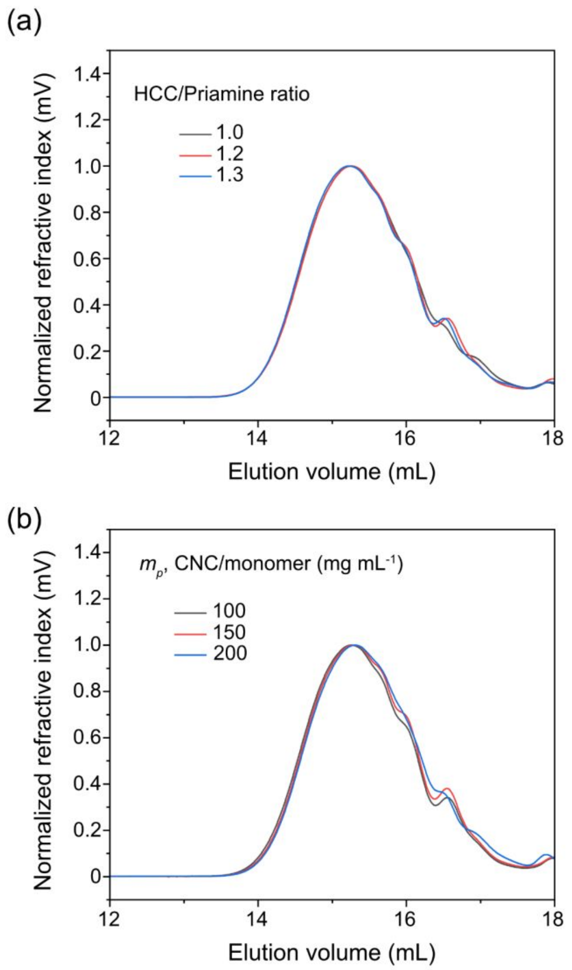


**Figure S2.** Effects of (a) HCC/Priamine ratio and (b) CNC concentration on molecular weight distribution of PHU latexes measured by SEC.

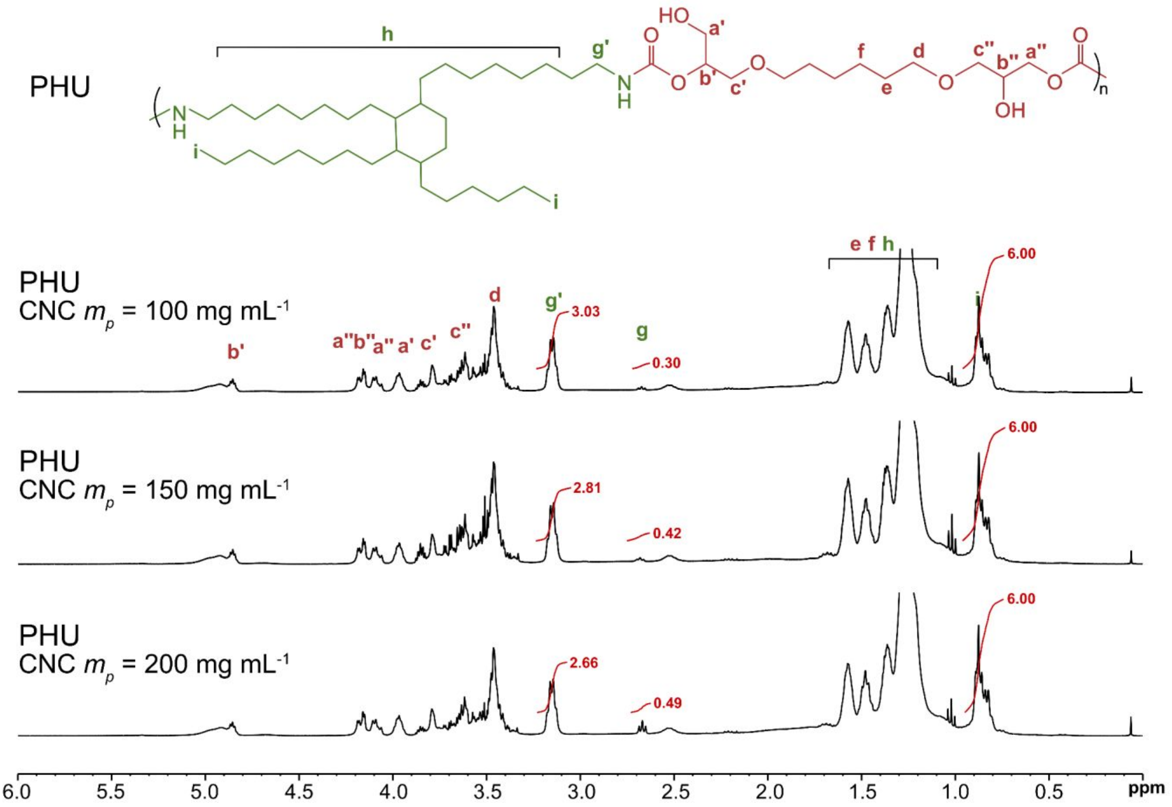


**Figure S3.** $^1$H NMR spectra of the PHU latex prepared using CNC concentrations ($m_p$) of 100, 150, and 200 mg mL$^{-1}$ at a fixed HCC/Priamine ratio of 1.2 using $CDCl_3$ as solvent.

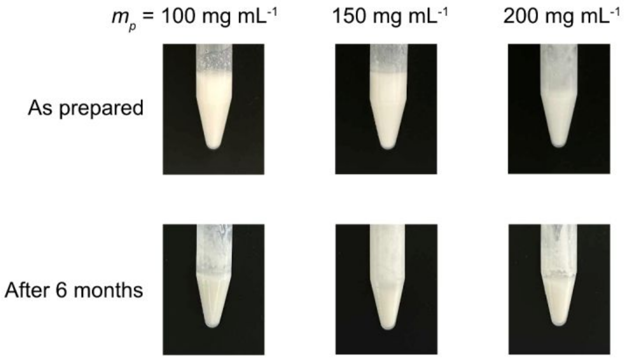


**Figure S4.** Appearance of PHU latexes prepared using CNC concentrations ($m_p$) of 100, 150, and 200 mg mL$^{-1}$, shown immediately after preparation and after 6 months of storage.

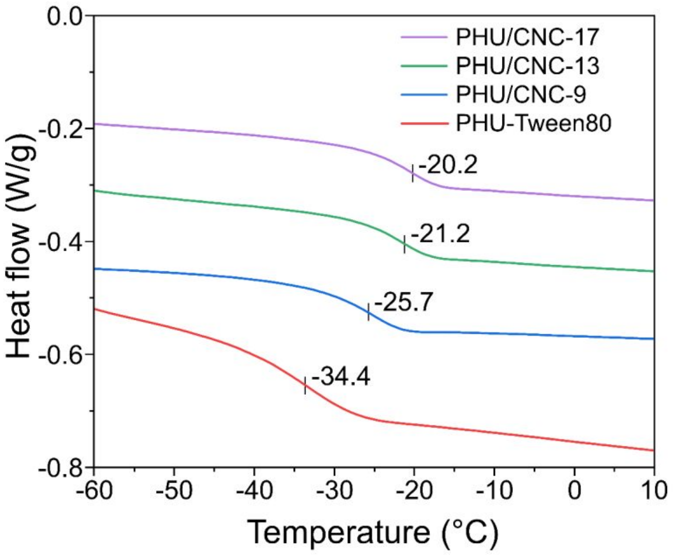


**Figure S5.** Second heating scans of DSC thermograms for Tween80-stabilized PHU and CNC-stabilized PHU nanocomposites with varying CNC contents (9, 13, and 17 wt %).

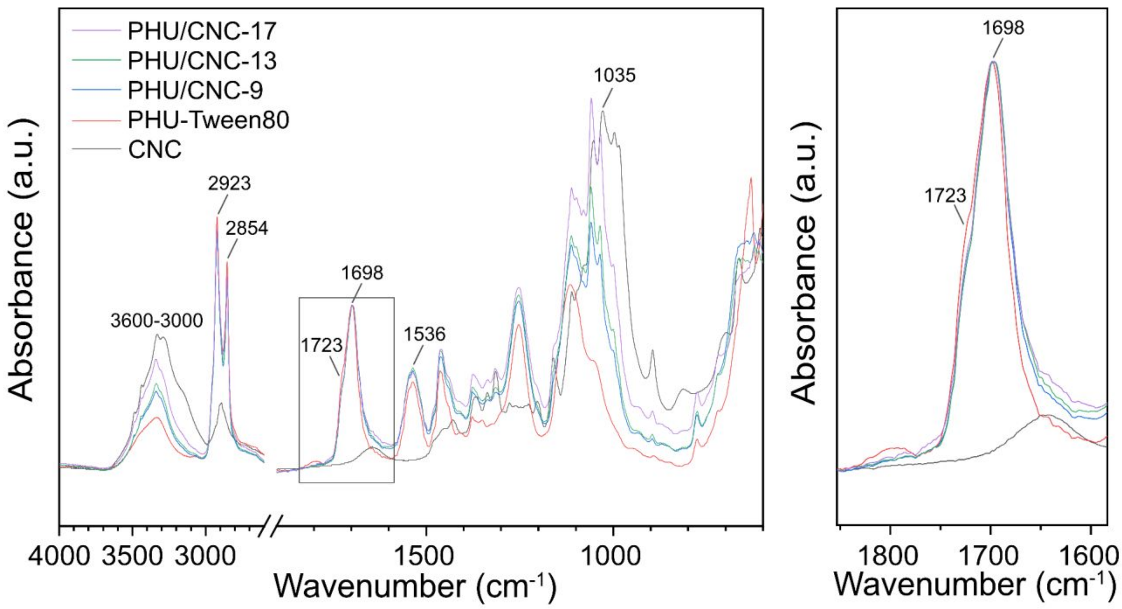


**Figure S6.** ATR-FTIR spectra of CNCs, Tween80-stabilized PHU (PHU-Tween80), and CNC-stabilized PHU nanocomposites containing 9, 13, and 17 wt % CNC. Spectral regions highlighting the carbonyl stretching bands are shown on the right for clarity

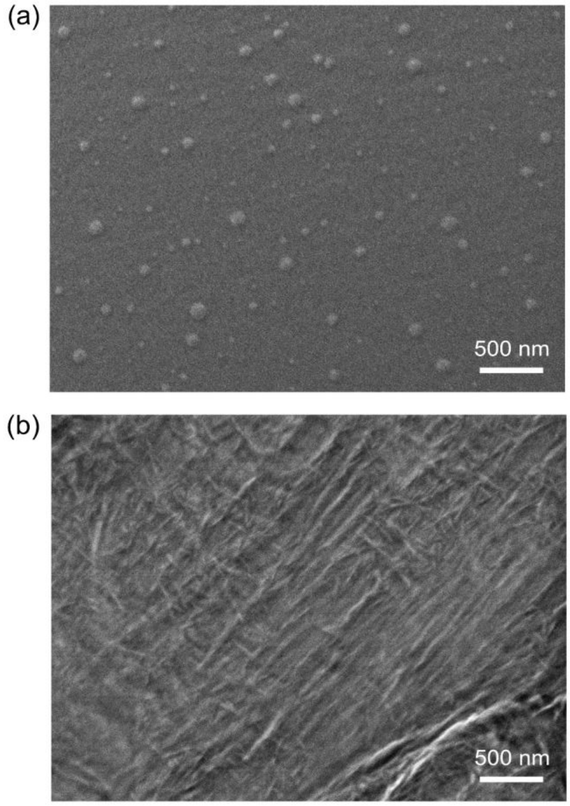


**Figure S7.** FESEM surface images of (a) Tween80-stabilized PHU and (b) PHU/CNC-17 nanocomposite at lower magnification.

**Table S1.** Summary of probe tack properties and $T_g$ of surfactant-stabilized PHU and CNC-stabilized PHU nanocomposites.

| Sample | $\sigma_{max}$ (kPa) | $W_{adh}$ (J m$^{-2}$) | $\varepsilon_{Max}$ | $T_g$ |
|---|---|---|---|---|
| PHU-Tween80 | 96.1 ± 11.0 | 8.0 ± 3.0 | 1.6 ± 0.2 | –34.4 |
| PHU/CNC-9 | 418.7 ± 25.0 | 33.4 ± 8.4 | 2.2 ± 0.2 | –25.7 |
| PHU/CNC-13 | 636.3 ± 59.5 | 39.9 ± 3.7 | 2.3 ± 0.1 | –21.2 |
| PHU/CNC-17 | 749.1 ± 11.3 | 46.4 ± 4.0 | 2.7 ± 0.4 | –20.2 |
